# Effect of Microencapsulation Shear Stress on the Structural Integrity and Biological Activity of a Model Monoclonal Antibody, Trastuzumab

**DOI:** 10.3390/pharmaceutics3030510

**Published:** 2011-08-24

**Authors:** Ritesh M. Pabari, Benedict Ryan, Catherine McCarthy, Zebunnissa Ramtoola

**Affiliations:** School of Pharmacy, Royal College of Surgeons in Ireland, 123 St. Stephens Green, Dublin 2, Ireland

**Keywords:** trastuzumab, shear stress, stability, size exclusion chromatography, circular dichroism, biological activity

## Abstract

The aim of the present study was to investigate the influence of process shear stressors on the stability of a model monoclonal antibody, trastuzumab. Trastuzumab, at concentrations of 0.4–4.0 mg/mL, was subjected to sonication, freeze-thaw, lyophilisation, spray drying and was encapsulated into micro- and nanoparticles. The stressed samples were analysed for structural integrity by gel electrophoresis, SDS-PAGE, and size exclusion chromatography (SEC), while the conformational integrity was analysed by circular dichroism (CD). Biological activity of the stressed trastuzumab was investigated by measuring the inhibition of cell proliferation of HER-2 expressing cell lines. Results show that trastuzumab was resistant to the process shear stresses applied and to microencapsulation processes. At the lowest concentration of 0.4 mg/mL, a low percent (<9%) of soluble/reversible aggregates may have been formed. No loss of structural integrity, conformation was observed and no significant change in the biological activity of trastuzumab was observed (ANOVA; *p* > 0.05). The results of this study conclude that trastuzumab may be resistant to various processing stresses. These findings have important implications with respect to pharmaceutical processing of monoclonal antibodies.

## 1. Introduction

In recent years a number of new monoclonal antibody based therapeutics have reached the market for the treatment of diseases such as cancer and immune-mediated diseases and many more are under development [1]. These monoclonal antibodies (mAbs) while having very potent therapeutic activity exhibit instability related to their structure. This poses various difficulties in their formulation, processing, storage and administration. It is extensively reported that these protein based therapeutics are labile molecules which can be structurally disrupted when exposed to various physical, thermal or chemical stressors [2-5]. Physical disruption of these molecules may manifest as denaturation [6] or aggregation [7] and is a common problem which may occur during manufacture, storage and administration of proteins including mAbs. Aggregates can form when these molecules are subject to various types of agitation or shear as a result of protein exposure to hydrophobic surfaces or air/water interfaces [8]. Aggregates formed can be reversible or irreversible and can have reduced activity or are inactive and may be immunogenic compared to the native molecule [9].

The anti-cancer monoclonal antibody cituximab has been shown to aggregate when subjected to stirring stress in a chemically independent manner [10]. Stathopulos *et al*, 2004 [11] reported that sonication induced the formation of aggregates that resemble amyloid in certain proteins. Aggregates can also form as a result of exposure to low or high temperatures. The exposure of a monoclonal antibody to freeze-thawing may result in aggregate formation via a number of mechanisms such as pH mediated conformational changes and adsorption to the ice-liquid interface [12,13]. An increase in temperature can also increase protein aggregation via chemical and non-chemical processes [14,15]. As a result of their sensitivity to agitation including rapid expulsion from a syringe, the reconstitution of mAbs is often recommended by manufacturers to be by gentle swirling and not shaking.

Monoclonal antibodies may also be subject to structural disruption by chemical means such as disulfide formation/exchange, oxidation or fragmentation [16]. Structural disruption may result in a marked reduction or indeed complete loss of biological activity and in addition may cause immunogenic type reactions [17,18].

Encapsulation of monoclonal antibodies into micro- and nanoparticulate delivery systems has stimulated much interest in the scientific literature [19]. The benefits of such delivery systems include protection from degradation, controlled release and ability to target to specific cells and tissues. In addition nanoparticulates have the ability to exploit the enhanced permeability and retention effect which occurs in solid tumors leading to elevated drug concentrations at the tumor site when compared with non-encapsulated drugs [20,21]. These nanoparticulate systems are currently being studied for their application to deliver monoclonal antibodies in the treatment of cancer as they offer multiple benefits to the patient. A limitation of these systems is that their manufacture exposes the therapeutic agent to various stressors which may be deleterious to the active, in particular to biological actives, such as proteins including monoclonal antibodies and may render the biological therapeutics inactive and/or possibly immunogenic.

Formulation of nanoparticulate systems exposes these biological molecules to one or more types of shear stress including sonication, homogenization and lyophilisation and these may disrupt the molecule resulting in the loss of its binding ability and biological activity. While the encapsulation of mAbs in such nanoparticulate systems have been studied, the effect of their exposure to the stresses involved has not been examined in detail. In this study we therefore investigated the effect of various shear stresses which commonly occur during the formulation and processing of particulate drug delivery systems, both individually and collectively, on the structural integrity and biological activity of trastuzumab (tzmAb), a model monoclonal antibody. Trastuzumab (Herceptin®), a recombinant humanized IgG1 monoclonal antibody (mAb), is used in the treatment of HER2 over-expressing breast cancers. Trastuzumab binds to the extracellular domain of the HER2 receptor and exerts its clinical action through inhibition of intracellular signaling via both the mitogen-activated protein kinase (MAPK) and phosphatidylinositol 3 kinase (PI3K) pathways [22,23]. Disruption of the molecule can affect its binding and hence its biological activity.

The stressors we examined included sonication, lyophilisation and freeze-thawing. In addition the effect of spray drying, a technique which is commonly used in the formulation of microparticles for drug delivery by routes such as inhalation was investigated. To study the effect of multiple stressors on the stability of the mAb, micro- and nanoparticles containing trastuzumab were subsequently prepared using spray drying and solvent evaporation from a double emulsion respectively and the encapsulated trastuzumab was analysed for structural integrity and biological activity.

## 2. Experimental Section

### 2.1. Materials

Reconstituted and diluted trastuzumab (Herceptin®; Roche Products Ireland Ltd.) solutions containing 0.4–4 mg/mL trastuzumab were a gift from Fannin Compounding Ireland Ltd. Poly-lactide-co-glycolide (PLGA, RG504H) polymer polymer was purchased from Boehringer, Ingelheilm, Germany. Chitosan of medium molecular weight (240 kDa), polyvinyl alcohol (PVA) of molecular weight ∼30–70 kDa, were obtained from Sigma-Aldrich (Ireland). All other reagents and HPLC grade solvents used were purchased from Sigma-Aldrich (Ireland).

### 2.2. Application of Mechanical/Shear Stresses on Trastuzumab Solutions

Various process shear stresses were applied to trastuzumab solutions of concentrations 0.4 to 4 mg/mL as described below.

#### 2.2.1. Sonication

Trastuzumab solutions of 0.4 and 4.0 mg/mL were sonicated using a probe sonicator (Bransonic 2510 Sonicator, Branson Ultrasonics Corporation, Connecticut, USA) at 1 Watt for 1 min and 3 min. Additionally, the higher concentration of 4.0 mg/mL trastuzumab solution was sonicated at a higher intensity of 3 Watts for 1 min and 3 min.

#### 2.2.2. Freeze-Thaw Cycle

Trastuzumab solutions at concentrations of 0.4 and 4 mg/mL were subjected to freeze thawing cycles by freezing each solution at −80 °C for 50 min and thawing at 4 °C. This cycle was repeated up to 6 times for each concentration studied.

#### 2.2.3. Lyophilisation (Freeze-Drying)

Trastuzumab solution at a concentration of 2mg/mL was lyophilized using the following protocol: Solutions were first frozen at −80 °C and subsequently freeze dried using a FreeZone 6 Liter Benchtop freeze drier (model no. 7752030, Labconco, Missouri, USA) operating at −56 °C and 0.036 mbar vacuum pressure. The samples were removed after 18 h and stored in the fridge at 4 °C until analysis.

#### 2.2.4. Spray Drying

Trehalose dihydrate was added to the trastuzumab solutions at a weight ratio of 1:1 and resultant solutions were spray-dried using a Mini Spray Dryer B-290 (Buchi, Switzerland) at an inlet temperature of 170 °C, aspirator rate of 80%, and a feed-flow rate of 4 mL/min. Air was used as the drying medium and the outlet temperature recorded was 76 °C.

### 2.3. Preparation of Nano- and Microparticles Containing Trastuzumab

Trastuzumab containing PLGA nanoparticles were prepared by a double emulsion solvent evaporation method adapted from Ramtoola *et al.*, [24]. Trastuzumab (15 mg) was dissolved in 1% w/v aqueous PVA and was added to 135mg of PLGA in ethyl acetate and sonicated at 1 Watt for 1 min to form a water-in-oil (w/o) emulsion which was then emulsified in 2.5% w/v aqueous PVA solution to form a double water-in-oil-in-water (w/o/w) emulsion using sonication at 1 watt for 1 min. The w/o/w emulsion was added to 1% w/v aqueous PVA and homogenised using an Ultra Turrax high speed homogeniser (TP 25, Janke-Kunkel, Staufen, Germany) operating at a speed of 13,500 rpm for 3 min. The emulsion formed was added to the deionised water and stirred for 8 h to allow solvent evaporation. The nanoparticles were recovered by centrifugation (Rotina 35 R, Hettich Zentrifugen, Tuttilingen, Germany) washed with deionised water to remove residual PVA and lyophilised as per lyophilisation protocol in section 2.2. Nanoparticles were stored at 4 °C until required.

Trastuzumab-containing chitosan microparticles at a theoretical trastuzumab loading of 5.6% w/w of chitosan were prepared as described previously [24]. Briefly, Trastuzumab solution containing d-(+)-trehalose dihydrate at a weight ratio of 1:1 was added to a solution of chitosan in 1% v/v aqueous acetic acid. The trastuzumab/chitosan solution was spray dried at a feed flow rate of 4 mL/min and an inlet temperature of 170 °C. The outlet temperature was monitored during the spray drying process and was found to be 83 °C. Nanoparticles formulated were characterised for their size using a zetasizer (Nanoseries, Nano-ZS; Malvern Instruments) while microparticles were sized using a mastersizer (Malvern Instruments, Malvern, Worcestershire, UK). The surface morphology of the particles was examined by scanning electron microscopy (Hitachi Scanning Electron Microscope (model S 3500N), Tokyo, Japan).

Trastuzumab from the nanoparticles was extracted by adding 0.5 mL ethyl acetate, followed by the addition of DI water. Samples were vortexed at 1400 rpm for 10 min, centrifuged at 11000 rpm for 5 min to form a two phase solution. The aqueous layer was removed by syringe for assay of drug content using the bicinchoninic assay kit (Pierce Thermo Scientific, Ireland) and analysis of structural integrity and bioactivity of trastuzumab.

### 2.4. Analysis of Trastuzumab for Structural and Conformational Integrity

Trastuzumab solutions before and after being subjected to the various mechanical stresses and trastuzumab extracts from micro and nanoparticles were analysed for structural or conformational changes using the methods described below.

#### 2.4.1. Sodium Dodecyl Sulfate-Polyacrylamide Gel Electrophoresis (SDS-PAGE)

SDS-PAGE was carried out using precast NuPAGE® Novex 4–12% Bis-Tris 1.0 mm Gel on XCell SureLock under reducing and non-reducing conditions as per the protocol from Invitrogen, CA, USA [25]. Approximately 1 μg (10 μL) of trastuzumab was loaded per well. The gel was stained with Coomassie Brilliant Blue R-250 staining solution (Bio-Rad, CA, USA) at 37 °C for 1 h in a shaker at 100 rpm and later destained with destaining solution (Bio-Rad, CA, USA) for up to 3 h at 37 °C.

#### 2.4.2. Size Exclusion Chromatography (SEC)

SEC-HPLC analysis as described previously by Tang *et al.* [26] was used to measure the structural integrity of trastuzumab samples and the trastuzumab concentration present in the samples. SEC-HPLC was conducted using a BioSep SEC S 3000 (Phenomenex, UK) column with a Perkin Elmer Series 200 analytical system (USA) with UV detection at 280 nm. The mobile phase consisted of 100 mM potassium phosphate, KH_2_PO_4_ (Sigma-Aldrich, Mo, USA), adjusted to pH 7.0 with 1 M NaOH (Merck, NJ, USA) and was run at a flow rate of 0.8 mL/min. The column was maintained in an oven at a temperature of 30 °C. Control trastuzumab solutions at a range of concentrations of 0.2 to 4 mg/mL were analysed to give a calibration curve which was used to determine concentrations of trastuzumab in the samples studied. Reproducibility of injections (*n* = 6) was carried out at 0.4, 1 and 4 mg/mL trastuzumab concentration. Control solutions and stressed samples were analysed in triplicate.

#### 2.4.3. Circular Dichroism (CD)

Circular dichroism (CD) measurements were performed using an Aviv CD Spectrophotometer 410 (Aviv Biomedical, Inc. New Jersey, USA). The stability of the secondary structure of trastuzumab for control trastuzumab solutions and trastuzumab samples at a concentration of 0.2 mg/mL in saline was probed using a quartz cell of 0.1 cm path length. The CD spectra for trastuzumab were collected in the far-UV region (195 to 260 nm). After an accumulation of 5 scans at a scan rate of 1 nm per second, the scans were corrected by subtracting the blank.

### 2.5. In Vitro Bioactivity Assay for Trastuzumab

Trastuzumab bioactivity was determined by measuring the inhibition in cell proliferation of SK-BR3 cells (passage 90), a human breast cancer cell line which over-expresses HER-2 as per the method described by Ozbay *et al.* 2010 [27]. SK-BR3 cells were grown in RPMI-1640 medium with 10% HI-FBS (BioSera UK) and passaged once per week. The cells were seeded in 96-well plate (Sarstedt, USA) at a density of 1 × 10^4^ cells/well. After the cells formed a monolayer at approximately 38 h, the medium was replaced by trastuzumab standards and samples of various concentrations diluted to give a dose response curve. The cells were incubated for 72 h after which 100 μL of 3-(4,5-dimethylthiazol-2-yl)-5-(3-carboxymethoxyphenyl)-2-(4-sulfophenyl)-2*H*-tetrazolium (MTS) reagent was added to each well and the plate incubated for 3 h. Absorbance was then read at 490 nm and converted to % inhibition of cell proliferation. The results are presented as log dose of trastuzumab (ug/mL) *vs.* % inhibition of cell proliferation (expressed as % of maximum trastuzumab (2mg/mL) mediated inhibition). Untreated cells (100 μL complete growth medium containing 10% FBS and 100 μL saline) were set at 100% proliferation.

## 3. Results and Discussion

### 3.1. Effect of Mechanical/Shear Stress on Structural Integrity of Trastuzumab

#### 3.1.1. Effect of Sonication

Sonication is a commonly used technique for enhancing dissolution rate, to deaggregate particles as well as to disrupt cell membranes or fragment DNA. It is also used in the formation of single or double emulsions during the formulation of biodegradable nanoparticles by the solvent evaporation process to encapsulate numerous therapeutic agents including proteins [28]. Sonication produces high frequency sound waves and has been reported to cause aggregation of proteins with molecular weight ranging from 13.7–66.4 kDa [11].

**Figure 1.**
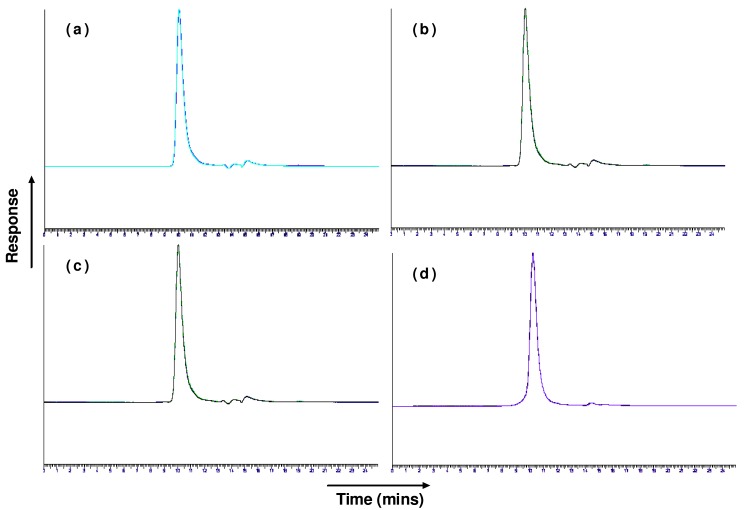
Size exclusion chromatography (SEC) chromatogram of native (control; light blue) trastuzumab overlayed with **(a)** 0.4 mg/mL trastuzumab sonicated at 1 Watt 1 min **(b)** 4.0 mg/mL trastuzumab sonicated at 1 Watt 1 min **(c)** trastuzumab freeze-thawed and **(d)** tratsuzumab freeze-dried.

**Figure 2.**
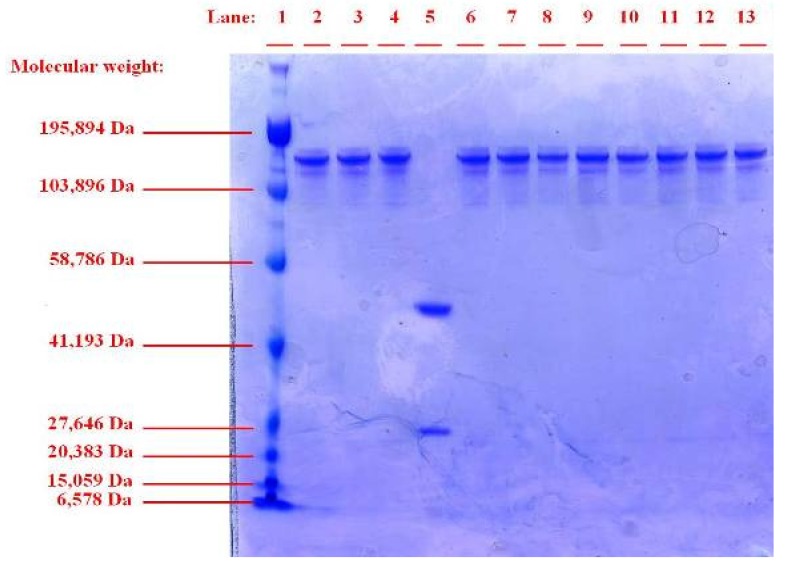
SDS-PAGE showing: molecular weight ladder (Lane 1), native trastuzumab (Lanes 2–4), chemically reduced trastuzumab (Lane 5), spray-dried trastuzumab (Lane 6), trastuzumab freeze-thawed x 6 cycles (Lanes 7–8), trastuzumab sonicated at; 1 Watt × 1 min (Lane 9), 3 Watt × 1 min (Lane 10), at 1 Watts × 3 min (Lanes 11-12) and trastuzumab extracted from Poly-lactide-co-glycolide (PLGA) nanoparticles (Lane 13).

In our study, sonication was not found to have any detrimental effect on trastuzumab structural integrity as was determined by SEC-HLPC and SDS-PAGE (Figures 1 and 2). No aggregation or degradation was observed when trastuzumab solution of 0.4 or 4 mg/mL was sonicated at low or high intensity of 1 and 3 watts. Increasing the exposure time of sonication at 1 Watt from 1 to 3 min for the trastuzumab 4 mg/mL solution showed no degradation or formation of soluble aggregate as a single band, corresponding to the molecular weight of ∼150 kDa for native trastuzumab when analysed by SDS-PAGE. When reduced chemically using dithiothreitol, trastuzumab was fragmented into its subunits of ∼25 kDa and ∼50 kDa representing the light and heavy chains of trastuzumab monomer respectively (Figure 2, lane 5).

No change in retention time, additional peaks or significant decrease in trastuzumab monomer concentration was observed by SEC analysis (Table 1 and Figure 1 a,b). No soluble/reversible aggregates were detected by SEC or SDS-PAGE. Exposure of trastuzumab at low and high concentration of 0.4 and 4 mg/mL had no impact on the structural integrity of trastuzumab irrespective of sonication intensity or duration of exposure.

**Figure 3.**
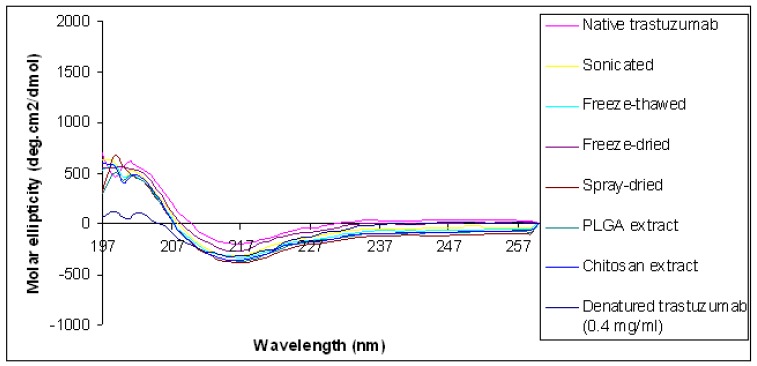
Circular dichroism (CD) spectra analysis of trastuzumab subjected to various mechanical stresses.

Circular dichroism was used to assess changes to the secondary structural conformation of trastuzumab following sonication. The circular dichroism (CD) spectra of the native trastuzumab showed an intensity that equals zero at 206 nm, a minimum at 217 nm and a broad shoulder around 230 nm indicating that the β-sheet is the most dominant secondary structure present (Table 2 and Figure 3). These results were consistent with that reported by Veermeer *et al.*, [5], for the native IgG molecule. Trastuzumab sonicated at the highest intensity of 3 Watts for 3 min showed no change in the wavelength at zero intensity, minima or broad shoulder when compared with the spectrum observed for native trastuzumab. Therefore, sonication of trastuzumab does not lead to the change in the secondary structural conformation of trastuzumab confirming that trastuzumab remains structurally and conformationally intact when exposed to sonication stress at the intensity and duration mentioned.

#### 3.1.2. Effect of Freeze-Thaw Cycle

During freezing, pH changes due to crystallisation of buffer components can occur and can result in the denaturation, aggregation and precipitation of proteins. In addition during freezing, new ice solution interfaces are created which increase the potential for aggregation and denaturation. Pikal-Cleland *et al.*, [29] reports irreversible damage to the structure, conformation and biological activity of both monomeric and tetrameric β-galactosidase in sodium phosphate buffer as a result of a decrease in pH during the freezing stage of freeze thawing experiments. Barnard *et al.*, [30] showed that when IgG2 solution in PBS at a pH 7.0 was frozen and thawed from −20 °C to room temperature for three cycles, insoluble aggregates were formed.

In our study exposure of trastuzumab to freeze-thawing had no detrimental effect on trastuzumab structural integrity and no soluble or insoluble aggregates were observed by SEC and SDS-PAGE (Figures 1c and 2). The SEC analysis of 0.4 mg/mL trastuzumab exposed to one, three or six freeze-thaw cycles showed a small decrease (<9%) in the monomer peak although no change in retention time or presence of additional peaks was observed when compared to the native trastuzumab (Table 1 and Figure 1c). It is possible that some soluble or reversible aggregates were formed at this low concentration but were below the detection limit of SEC and SDS-PAGE. The structural integrity of trastuzumab at the higher concentration of 4 mg/mL was maintained after exposure to freeze thaw with little or no loss in monomer concentration. Trastuzumab at 0.4 and 4 mg/mL subjected to six freeze-thaw cycles showed one band corresponding to the native trastuzumab and no additional bands on SDS-PAGE gel was observed (Figure 2). The conformational integrity of trastuzumab after exposure to six freeze thaw cycles was confirmed by CD analysis as no change in its spectrum was observed when compared to that of native trastuzumab (Table 2 and Figure 3).

#### 3.1.3. Effect of Lyophilisation (Freeze-Drying)

Lyophilisation, or freeze-drying, is a commonly used method for the preservation of labile materials such as proteins in a dehydrated form. Freeze-drying is also a common recovery method for protein encapsulated in nanoparticles. Lyophilisation is generally carried out in the presence of a cryoprotectant such as sucrose, trehalose or sorbitol to immobilize the protein in a glassy matrix and maintain the bioactivity of the protein molecules.

Lyophilisation of trastuzumab solutions showed no decrease in the monomer concentration, when compared to native trastuzumab (Table 1 and Figure 1d). Similarly, when analysed by CD no significant differences in the CD spectrum was observed when compared to the native trastuzumab (Table 2 and Figure 3) confirming no conformational changes of the trastuzumab molecule occurred when lyophilised. Trastuzumab is marketed as the freeze-dried form ready for re-constitution. When lyophilised after reconstitution and diluted in saline, no detrimental effect on trastuzumab structural integrity was observed. The presence of additional sodium chloride in the reconstituted and diluted trastuzumab solutions did not adversely affect the trastuzumab during freezing/drying process.

#### 3.1.4. Effect of Spray Drying

Spray drying is commonly utilized in the drying of actives and excipients in the pharmaceutical industry. It is also extensively used for granulation and microencapsulation. Spray drying of biological molecules is often associated with potential instability of these molecules as a result of their exposure to the high temperatures used. In addition the proteins are subject to considerable shear forces due to the spraying mechanism as the solutions are fluidised through a fine nozzle to form the droplets in contact with drying air or nitrogen. Chen *et al.* [31] reported that antibodies are vulnerable to denaturation via thermal stress. Adler *et al.* [32] showed that the stability of the model protein lactate dehydrogenase (LDH) during spray-drying was a function of the spray-drying temperature and drying time and was related to the presence of the protein at the liquid/air interface. Adler *et al.* [32] reported that the addition of suitable surfactant such as polysorbate 80 can prevent the LDH protein from appearing at the surface of the droplets and hence prevent protein inactivation.

Spray drying of trastuzumab solution was performed at an inlet temperature of 170 °C and a flow rate of 4 mL/min using heated air as drying medium. Interestingly, no detrimental effect on trastuzumab structural integrity was observed when analysed by SDS-PAGE and CD (Lane 6, Figure 1 and Figure 3). No difference between the spray dried sample was found compared to the native trastuzumab, reflecting no aggregation, degradation or change in the trastuzumab conformation. Vermeer *et al.* [5] reported that, at temperatures above 65 °C, the wavelength at zero intensity shifts to a lower value of less than 200 nm, compared to 206 nm for the native trastuzumab and this is indicative of a less ordered structure. In our study, the wavelength at zero intensity was at 206 nm for the spray dried trastuzumab and was 207 nm for the native trastuzumab. When trastuzumab solution was incubated at a temperature of 75 °C a shift of the wavelength at zero intensity from 207 nm to 204 nm was observed (Figure 3 and Table 2) indicating a shift to a less ordered structure of the trastuzumab at the incubation temperature. The stability of trastuzumab at the high spray drying temperature of 170 °C is probably due to the short exposure time, of seconds, of the mAb to this elevated temperature. In addition trehalose which is used as a cryoprotectant during lyophilisation of proteins, was added to the tratsuzumab solution and this may have acted as a protectant during spray drying.

The structural stability of each monoclonal antibody varies with respect to its resistance to stress, for example it has been reported that trastuzumab when subjected to temperatures of 70 degrees for 15 min did not lead to aggregation or decomposition [33]. It is possible therefore that trastuzumab has a higher resistance to both shear and temperature stressors.

### 3.2. Effect of Microencapsulation Shear and Temperature on the Structural Integrity of Trastuzumab

**Figure 4.**
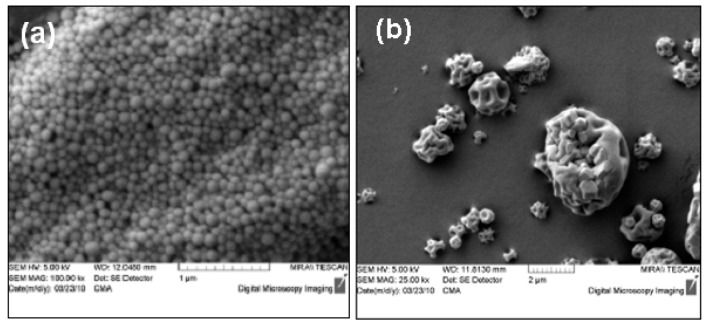
Scanning electron micrographs of trastuzumab containing **(a)** PLGA nanoparticles, **(b)** chitosan microparticles.

Trastuzumab containing nano and microparticles formulated showed similar particle size and morphology as previously reported for similar particulate formulations from our research group [24]. The nanoparticles had a diameter of 206.48 ± 0.60 nm and were smooth and spherical (Figure 4 a). The spray dried microparticles formed had a median diameter of 13.22 ± 0.73 μm and were spherical with a puckered surface (Figure 4 b).

The trastuzumab content of the nanoparticles was found to be 13.7 ± 1.7% w/w. The trastuzumab loading of the microparticles was in the range of 6.1–6.8% w/w.

The application of single stressors as described above was not found to affect the structural integrity of trastuzumab and did not result in aggregate formation. During micro/nanoparticle formulation, depending on the method used, the active can be subject to multiple successive stressors which can include sonication, homogenization, centrifugation, lyophilisation, contact with organic solvents as in the case of solvent evaporation from emulsion systems. When using spray drying as microencapsulation technique, the active is subject to shearing through a nozzle in addition to exposure to high temperature above 100 °C for aqueous solutions. Van de Weert *et al.* [34] reported that the use of an oil-in-oil emulsion may reduce potential denaturation stress when compared to the use of aqueous solutions as the outer phase.

In our study, a double emulsion, a water-oil-water (w/o/w) emulsion, was used to formulate particles containing trastuzumab using the biodegradable polymer PLGA dissolved in ethylacetate. The w/o/w emulsion was formed by sonication at 1 watt over a total time of 2 min followed by high shear homogenization at 13,500 rpm for 3 min. The emulsion was subsequently subject to centrifugation followed by lyophilisation.

SDS-PAGE analysis on the trastuzumab extracted from the nanoparticles showed a single band (Lanes 13, Figure 2) similar to that of native trastuzumab. No aggregate or fragmentation of the extracted trastuzumab was observed suggesting that structural integrity was maintained during the encapsulation of trastuzumab, irrespective of its exposure to multiple stressors. When examined by CD, the spectrum for the trastuzumab extracted from particles was found to be similar to that of the native trastuzumab (Figure 3) with no significant shift at any of the identified wavelength of zero intensity, minimum and shoulder (Table 2) showing the retention of the β-sheet dominance of the secondary structure and conformation.

Similarly, microencapsulation of trastuzumab with chitosan polymer by spray drying at 170 °C was not found to affect the structural integrity or secondary structure of trastuzumab. The findings in this study are surprising as proteins and in particular mAbs are generally considered labile molecules too fragile for exposure to the stressors examined in this study.

### 3.3. Evaluation of the Effect of Processing Stressors on the Biological Activity of Trastuzumab

While the results above have shown absence of aggregate formation, denaturation and degradation or fragmentation of trastuzumab, the effect on biological activity of the molecule is critical as it dictates its in vivo performance. Therefore the effectiveness of trastuzumab to inhibit cell proliferation of SK-BR3 cells, following its exposure to the various stressors was studied and was compared with that of native or control trastuzumab.

**Figure 5.**
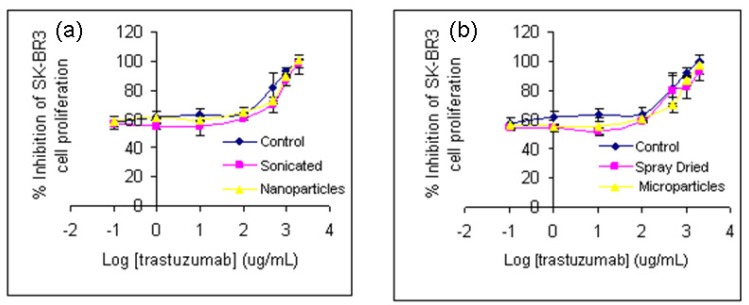
**(a)** The effect of sonication and nanoparticle formulation on the trastuzumab mediated inhibition of SK-BR3 cells. **(b)** The effect of spray drying and microparticle formulation on the trastuzumab mediated inhibition of SK-BR3 cells. Data shown as mean of *n* = 3 ± SD.

No significant difference in the biological activity of trastuzumab was observed when subjected to sonication, spray drying or after formulation as nano or microparticles (*p* > 0.05), One Way ANOVA, Figures 5 a,b). The exposure of trastuzumab to any of these stressors studied singly or in combination or its exposure to spray drying at elevated temperature of 170 °C therefore did not affect its biological activity. This supports our earlier observations that no loss of structure or conformation of trastuzumab occurred when exposed to one or more of the physical/thermal stress studied.

## 4. Conclusions

Instability is an inherent characteristic of protein molecules which impacts on the processing conditions and techniques used in their manufacture. This instability is often a barrier to their further development as micro- and nanoparticles for controlled and targeted delivery. In this study, we examined the effect of single or multiple shear/thermal stresses commonly encountered in pharmaceutical and microencapsulation processes on a model mAb, trastuzumab. Our results showed that trastuzumab was less vulnerable to shear/thermal processing conditions than was expected. Trastuzumab was not adversely affected by exposure to any of the stressors either singly or in combination. The presence of trehalose probably acted as a stabiliser for tratsuzumab during spray drying. According to our results, it appears that trastuzumab may be a protein which is resistant to physical stresses and to short exposure to thermal stresses. It is possible that other mAbs may be similarly less vulnerable to certain stresses. Interestingly, the findings in this study also suggest that it may be possible to utilise other drying technologies such as spray drying besides lyophilisation in their manufacture. The potential of spray drying for the formulation and manufacture of therapeutic peptides and proteins therefore warrants further consideration.

## Figures and Tables

**Table 1.** Concentration of trastuzumab monomer as determined by size exclusion chromatography (SEC) after subjected to various mechanical stress. Data shown as mean of n = 3 ± SD.

**Sample/stressed sample**	**Level of stress**	**Assayed trastuzumab monomer (mg/mL)**	**(%) original trastuzumab monomer**
Trastuzumab (0.4 mg/mL)	None	0.38 ± 0.01	100.0
Trastuzumab (2 mg/mL)	None	1.94 ± 0.03	100.0
Trastuzumab (4 mg/mL)	None	3.95 ± 0.04	100.0
Sonicated (0.4 mg/mL)	1 Watt, 1 min	0.38 ± 0.02	100.6 ± 6.6
Sonicated (4 mg/mL)	1 Watt, 1 min	3.94 ± 0.05	99.8 ± 1.2
Freeze-thaw (0.4 mg/mL)	1 cycle	0.36 ± 0.01	94.8 ± 3.2
Freeze-thaw (0.4 mg/mL)	3 cycles	0.35 ± 0.00	94.3 ± 1.0
Freeze-thaw (0.4 mg/mL)	6 cycles	0.34 ± 0.02	91.3 ± 5.1
Freeze-thaw (4 mg/mL)	1 cycle	3.94 ± 0.02	99.8 ± 0.4
Trastuzumab (2 mg/mL)	lyophilized	1.89 ± 0.01	97.3 ± 0.5

**Table 2.** Circular dichroism (CD) analysis of trastuzumab subjected to various mechanical stresses.

**Trastuzumab samples**	**Wavelength at zero intensity**	**Spectra minima**	**Broad shoulder**
Native trastuzumab	207 nm	216 nm	235 nm
Sonicated at 3 Watts for 3 min	206 nm	216 nm	236 nm
Freeze-thaw by 6 cycles	206 nm	215 nm	234 nm
Freeze-dried	206 nm	215 nm	236 nm
Spray-dried	206 nm	215 nm	234 nm
Extract from PLGA nanoparticles	206 nm	217 nm	235 nm
Extract from chitosan microparticles	206 nm	217 nm	235 nm
Denatured Trastuzumab	204 nm	215 nm	230 nm
